# Brain condition may mediate the association between training and work engagement

**DOI:** 10.1038/s41598-020-63711-3

**Published:** 2020-04-22

**Authors:** Keisuke Kokubun, Yousuke Ogata, Yasuharu Koike, Yoshinori Yamakawa

**Affiliations:** 1 Office of Society-Academia Collaboration for Innovation, Kyoto University, Kyoto, Japan; 2 Institute of Innovative Research, Tokyo Institute of Technology, Meguro, Tokyo Japan; 3 ImPACT Program of Council for Science, Technology and Innovation (Cabinet Office, Government of Japan), Chiyoda, Tokyo Japan; 4 Office for Academic and Industrial Innovation, Kobe University, Kobe, Japan; 5 NTT Data Institute of Management Consulting, Inc., Kobe, Japan

**Keywords:** Motivation, Social behaviour

## Abstract

Over the past two decades, the number of studies on work engagement has increased rapidly. Work engagement refers to a positive, affective-motivational state of high energy combined with high levels of dedication and a strong focus on work, leading to various work-related outcomes, including higher work performance. Several studies have indicated that training or coaching may increase work engagement, but other studies have shown contradicting results. These inconsistencies may be due to the indirectness between training/coaching and work engagement. Therefore, we investigated the relationship between training and brain structure as well as between brain structure and work engagement in cognitively normal participants. Brain structure was assessed using neuroimaging-derived measures, including the gray-matter brain healthcare quotient (GM-BHQ) and the fractional-anisotropy brain healthcare quotient (FA-BHQ), which are approved as the international standard (H.861.1) by ITU-T. Work engagement was assessed using the Utrecht Work Engagement Scale. To validate and enrich the analysis, we employed another two representative questionnaires, which are known to be close to but different from work engagement: The Social interaction Anxiety Scale and the Maslach Burnout Inventory-General Survey to gauge the levels of human relation ineffectiveness and burnout. The latter scale is subdivided into three variables including “Exhaustion,” “Cynicism,” and “Professional Efficacy.” The results of the present study indicate that training is associated with an increase of FA-BHQ scores, and that an increase of the FA-BHQ scores is associated with an increase in Work Engagement and a decrease in Cynicism. On the other hand, the training with coaching was associated with a decrease in Interaction Anxiety. However, no correlation was observed for training with Work Engagement or the subscales of Burnout. Likewise, no correlation was observed for FA-BHQ with Exhaustion, Professional Efficacy, and Interaction Anxiety. The results of the current research provide the possibility to use brain information to evaluate training effectiveness from the viewpoint of neuroscience.

## Introduction

Over the past two decades, the number of studies on work engagement has increased rapidly. Work engagement refers to a positive, affective-motivational state of high energy combined with high levels of dedication and a strong focus on work^1^. Contemporary public and private organizations should have engaged employees because previous research suggests that high work engagement leads to work performance^2–6^, innovative behavior^7^, job satisfaction, lower turnover intention^8–12^, high levels of creativity, task performance, organizational citizenship behavior, client satisfaction^13^, greater profitability, shareholder returns, productivity, and customer satisfaction^14–16^.

The research into employee engagement has been informative and useful, but up until now has tended to focus solely on what organizations can do to engage their employees (e.g. training supervisors to be supportive to subordinates)^17,18^. However, although several studies showed a positive link between an individual’s experiences in a range of interventions, including new ways of working, forum theatre training and mindfulness training and engagement^19,20^, one other study found no change in engagement levels following a workload intervention exercise^21^ (see also a narrative review by Bailey and colleagues^8^). Likewise, although it was found that coaching has a positive relationship with work engagement^2,22^, decreased stress^23^, and decreased depression and anxiety^24^, MacKie^25^ questioned whether coaching always had a positive impact. In support of this hypothesis, a meta-analysis indicated that there was an inconsistent relationship between coaching and coaching outcomes^26^.

The inconsistency in the association between training/coaching and engagement may be due to indirectness. The former may be prepared by organizations, while the latter may be engendered as the outcome at an individual’s level. However, there has been little consideration to date of what can be done at the individual level to help people achieve the right mindset and attitude for engagement^27^. In other words, the cognitive or affective mechanisms within the human brain, the mediator between training and engagement, could influence the results of training; therefore, a direct and significant relationship between training and engagement might have been difficult to observe. If so, understanding the effect of training on brain condition, rather than on engagement, is critical for the determination of modifiable factors that can increase engagement by any possible intervention. Indeed, it has been indicated that cognitive ability is among the strongest predictors of training effectiveness^28^ because it helps to prioritize organizational goals^29^, engaging training, and performing the skills taught in the course^30–33^.

Regarding the relation between training and brain, using diffusion imaging, Scholz and colleagues^34^ detected a localized increase in fractional anisotropy (FA), a measure of microstructure, in the white matter underlying the intraparietal sulcus following training of a complex visuomotor skill, providing the first evidence for training-related changes in white-matter structure in the healthy human adult brain. On the other hand, Takeuchi and colleagues^35^ observed that after cognitive training, there was an increase in FA in the parietal and frontal cortices. Likewise, Tang and colleagues^36^ observed an increased FA in the anterior cingulate cortex after 11 hours of integrative body-mind training. In line with this, training on working memory updating, mental set shifting, episodic memory, and processing speed tasks produced an increase in the white matter microstructure of the anterior section of the corpus callosum^37^. More recently, Colom and colleagues^38^ compared a training group that played a cognitively complex commercial videogame with a control group, observing training-related increases in white matter integrity in the right hippocampal cingulum bundle and the left inferior longitudinal fasciculus.

However, to our knowledge, there has been no study investigating the relationship between human resource development training (HRDT) and brain health. Therefore, this study aimed to investigate the relationships between HRDT and brain structure as well as between brain structure and work engagement in cognitively normal participants. Also, we employed another two sets of scales, human relation and burnout, which are often discussed as an important theme in the workplace; The former is supposed to be a potential social factor that may influence work engagement, and the latter is considered a state opposing work engagement.

We deal with human relationships in this study because social support^39^ and psychological safety^40^ play a crucial role in work engagement. For instance, it is reported that people working in jobs with a high level of feedback, obtaining direct and clear information about the effectiveness of his or her performance^41^, tend to be more engaged^42,43^. Moreover, overall work engagement may change, dependent on personal resources^44^ or team members’ work engagement^45^. Similarly, the reason why we focus on burnout in this study is that  burned-out employees often find it difficult to satisfactorily complete their usual work assignments^46^. Bakker and Costa^47^ have suggested that employees with high burnout levels show self-undermining behavior by making more mistakes or creating more interpersonal conflicts at work. In lie with this, based on longitudinal designs, other researchers reported that burnout is a significant predictor of low work engagement^48^. Some discussions swapped cause and effect; Van Beek and colleagues^49^ considered that work engagement is rather an antecedent of low exhaustion, suggesting that highly engaged students are less vulnerable to exhaustion (a component of burnout), as compared with students with low engagement. Other research examined the relationships between work engagement, human relationships, and burnout. For example, it is reported that engagement and burnout show similar patterns of associations with many job characteristics including social supports from the supervisors^50^.

**Figure 1:**
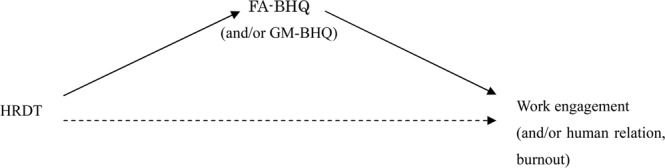
Expected association between human resource development training (HRDT), FA-BHQ (and/or GM-BHQ), and the work engagement (and/or human relation and burnout). Note: HRDT, Human resource development training; FA-BHQ, fractional-anisotropy brain healthcare quotient; GM-BHQ, gray-matter brain healthcare quotient.

We examined brain structure using neuroimaging-derived measures, including the gray-matter brain healthcare quotient (GM-BHQ) and the fractional-anisotropy brain healthcare quotient (FA-BHQ), which are approved as the international standard (H.861.1) by International Telecommunication Union Telecommunication Standardization Sector (ITU-T). We have found the possibility that these measures could be a means to objectively evaluate stress, fatigue^51^, food balance^52^, healthy lifestyles^53^, behavioral ambidexterity^54^, and cognitive ability^55^. Work engagement was calculated using the Utrecht Work Engagement Scale^56^; and human relation and burnout were assessed using the Social Interaction Anxiety Scale^57^ and the Maslach Burnout Inventory-General Survey^58^, respectively. The tested hypotheses are presented as follows: (1) HRDT correlates with higher FA-BHQ (and/or GM-BHQ) scores; (2) HRDT and the FA-BHQ (and/or GM-BHQ) scores independently correlate with work engagement (and/or human relation and burnout), and the FA-BHQ (and/or GM-BHQ) scores have a stronger association with work engagement (and/or human relation, burnout) than training. Figure 1 describes the possible association between HRDT, FA-BHQ (and/or GM-BHQ), and work engagement (and/or human relation, burnout).

## Materials and methods

### Participants

Eighty-two healthy participants (10 females and 72 males), aged 21–61 years (mean [M] ± standard deviation [SD]: 40.5 ± 9.2 years) were recruited in Tokyo, Japan. Potential participants with a history of neurological, psychiatric, or medical conditions that could affect the central nervous system were excluded from the study. However, “healthy” participants may include those who suffer burnout because burnout is not only difficult to be screened out except through questionnaire survey but also not officially recognized as a mental illness or mental disorder, and instead, it falls into the category of adjustment disorders^59^. This study was approved by the ethics committees of Kyoto University (approval number 27-P-13) and Tokyo Institute of Technology (approval number A16038) and was performed following the guidelines and regulations of the institute. All participants provided written informed consent before participation, and participant anonymity was preserved.

We used MRI to measure gray- and white-matter changes in a longitudinal study of individuals experiencing HRDT. We obtained an informed consent from 82 participants and placed them into either training group A (n = 16), training group B (n = 15), training group C (n = 12) or an untrained control group (n = 39). The participants were scanned before and after 3 months including 2-day to 8-week training periods depending on the kind of training.

Among them, sixteen participants in group A (1 female and 15 males, aged 21–59 years, mean [M] ± standard deviation [SD]: 39.2 ± 12.6 years) and fifteen participants in group B (2 females and 13 males, aged 28–54 years, mean [M] ± standard deviation [SD]: 40.1 ± 6.7 years) answered the common questionnaire we prepared on the same day and at the same place of the MRI experiments. Two weeks later, they underwent training for two days to understand the importance of establishing identities and mutual understanding and building related habits within their life, to progress from dependence through independence on to interdependence. Training consisted of textbook pre- and after-learning, video screenings, instructor commentary, Q & A sessions, and group discussions. Additionally, participants in group B received 30 minutes coaching for 4 times but those in group A did not. The coaching was held in 8 days for a person aiming to establish thinking and behavior change along the line of training. Three months after the 1st experiments, they were gathered to the same place to undergo the same MRI experiments and to answer the same questionnaire.

On the other hand, twelve participants in group C (3 females and 9 males, aged 33–61 years, mean [M] ± standard deviation [SD]: 42.6 ± 9.2 years) undertook a three-hour training regarding the importance of heightening happiness and bringing out positive feelings soon after the day of the MRI experiment. Then, the subjects dealt with two of ten subjects (e.g., writing out successes experienced and how they felt at those times) for six days, followed by follow-up trainings including making up collage images five years later. Three months after the 1st experiments, the subjects underwent MRI experiments again. All training (for groups A, B, and C) was conducted in cooperation with two reputable training companies in Japan and overseas. Besides, another thirty-nine participants (4 females and 35 males, aged 26–58 years, mean [M] ± standard deviation [SD]: 40.6 ± 8.6 years) were recruited in the control group and did nothing other than undertaking the MRI experiments (in common with groups A, B, and C) and answering a questionnaire (common with the group A and B).

### MRI data acquisition

All magnetic resonance imaging (MRI) data were collected using a 3-Tesla MRI scanner (MAGNETOM Prisma, Siemens, Munich, Germany) with a 32-channel head array coil at Tokyo Institute of Technology. A high-resolution structural image was acquired using a three-dimensional (3D) T1-weighted magnetization-prepared rapid-acquisition gradient echo (MP-RAGE) pulse sequence. The parameters were as follows: repetition time (TR), 1900 ms; echo time (TE), 2.52 ms; inversion time (TI), 900 ms; flip angle, 9°; matrix size, 256 × 256; field of view (FOV), 256 mm; slice thickness, 1 mm. DTI data were collected with spin-echo echo-planar imaging (SE-EPI) with GRAPPA (generalized autocalibrating partially parallel acquisitions). The image slices were parallel to the orbitomeatal (OM) line. The parameters were as follows: TR, 14100 ms; TE, 81 ms, flip angle, 90°; matrix size, 114 ×114; FOV, 224 mm; slice thickness, 2 mm. A baseline image (b = 0 s/mm^2^) and 30 different diffusion orientations were acquired with a b value of 1000 s/mm^2^.

### MRI data analysis

T1-weighted images were preprocessed and analyzed using Statistical Parametric Mapping 12 (SPM12; Wellcome Trust Centre for Neuroimaging, London, UK) running on MATLAB R2015b (Mathworks Inc., Sherborn, MA, USA). Here, SPM12 includes the preprocessing steps of segmentation, bias correction, and spatial normalization are incorporated into a single generative model. Using SPM12 prior probability templates, each MPRAGE image was segmented into GM, white matter (WM), and cerebrospinal fluid (CSF) images. To aid segmentation by correcting for smooth intensity differences that varied in space induced by the scanner, the intensity non-uniformity bias correction was applied. Subsequently, using the diffeomorphic anatomical registration through exponentiated lie algebra (DARTEL) algorithm^60^, the segmented GM images were spatially normalized. To reflect regional volume and preserve the total GM volume from before the warp, a modulation step was also incorporated into the preprocessing model. As a final preprocessing step, with an 8-mm full width at half-maximum (FWHM) Gaussian kernel, all segmented, modulated, normalized images were smoothed. by summing the GM, WM, and CSF images for each subject, intracranial volume (ICV) was also calculated. To control for differences in whole-brain volume across participants, proportional GM images were generated by dividing smoothed GM images by ICV. Mean and standard deviation (SD) images were generated from all participants using these proportional GM images. Next, we calculated the GM brain healthcare quotient (BHQ), where the mean value was defined as BHQ 100 and SD was defined as 15 BHQ points. Approximately 95% of the population is between BHQ 70 and BHQ 130 by this definition. The following formula, 100 + 15 × (individual proportional GM—mean) / SD, was used to calculate individual GM quotient images. Then, using an automated anatomical labeling (AAL) atlas^61^, regional GM quotients were extracted and averaged across regions to produce participant-specific GM-BHQs.

Using FMRIB Software Library (FSL) 5.0.9^62^, DTI data were preprocessed. First, with the initial b0 image, all diffusion images were aligned; and using eddy_correct, motion correction and eddy current distortion correction were  performed. FA images were calculated using dtifit, following these corrections. Then, using FLIRT and FNIRT, FA images were spatially normalized into the standard Montreal Neurological Institute (MNI) space (here, we smoothed the data with an 8-mm FWHM). After spatial normalization, mean and SD images were generated from all the FA images; and using the formula, 100 + 15 × (individual FA − mean)/SD, individual FA quotient images were calculated. Lastly, using Johns Hopkins University (JHU) DTI-based white-matter atlases^63^, regional FA quotients were extracted and averaged across regions to produce participant-specific FA-BHQs. For more details, please see Nemoto *et al*.^64^.

### Psychological scales

Work engagement was measured using the Utrecht Work Engagement Scale (UWES) developed by Schaufeli and colleagues^56^, which contains 17 items answered on a 7-point scale including “When I get up in the morning, I feel like going to work.” Although the original UWES may be subdivided into three scales (i.e., vigor, dedication, and absorption), we here use combined figures following the recommendation “at least in Japan—work engagement should be treated as a unitary construct” (Shimazu *et al*.: 519)^65^.

The Social Interaction Anxiety Scale (SIAS)^57^ contains 20 items answered on a 5-point Likert scale, including “I get nervous if I have to speak with someone in authority (teacher, boss, etc.).” On the other hand, the Maslach Burnout Inventory-General Survey (MBI)^58^, answered on a 7-point scale is subdivided into three variables including “Exhaustion,” “Cynicism,” and “Professional Efficacy.” “Exhaustion” contains five items including “I feel emotionally drained from my work.” “Cynicism” contains five items including “I have become less enthusiastic about my work.” “Professional Efficacy” contains six items including “I can effectively solve the problems that arise in my work.”

SIAS is used to assess distress when meeting and talking with other people including fears of being inarticulate, boring, sounding stupid, not knowing what to say or how to respond within social interactions, and of being ignored^57^. The reason why we employed SIAS is that  internal communications are necessary to effectively convey the values of an organization to employees to result in more engaged employees^66,67^. In line with this, it was found that well-designed internal communication training programs could be an important factor for employee engagement^66,68^. Therefore, we believed that observing SIAS, a variable well-related with work engagement could be a good reference to consider the link between training and brain and work engagement by observing its effect on the brain.

Burnout is a psychological syndrome that involves a prolonged response to chronic interpersonal stressors and leads to poor job performance, withdrawal behaviors and poor mental health^69^. The reason why we employed MBI in the current research is that it is often discussed that engagement may be assessed by determining the opposite pattern of scores on the three dimensions of MBI: low scores on exhaustion and cynicism, and high scores on professional efficacy^70,71^. Therefore, using MBI, we believe we could validate and examine the meaning of the association between training, brain, and work engagement. Moreover, if we could elucidate the link between the brain condition and burnout, we may enable timely preventive solutions before they become more serious and pervasive^72^.

### Statistical analysis

Regression analysis was employed to assess the correlation between training, ΔGM-BHQ (increase of GM-BHQ), ΔFA-BHQ (increase of FA-BHQ), and psychological scales. All statistical analyses were performed using IBM SPSS Statistics Version 26 (IBM Corp., Armonk, NY, USA).

## Results

Table 1 indicates that there was no statistical mean difference among the four groups for GM-BHQ (*F* (3, 78) = 0.183, p = 0.908), FA-BHQ (*F* (3, 78) = 0.829, p = 0.482), Age (*F* (3, 78) = 0.315, p = 0.815), and BMI (*F* (3, 78) = 0.478, p = 0.699) according to the analysis of variance (ANOVA). Likewise, there was no statistical distributional difference in sex (χ^2^ (3) = 2.521, p = 0.472) according to the results of the chi-square test. Additionally, there was no statistical difference among the groups for the five psychological scales. Therefore, it is safe to say that the distribution of research participants was homogeneous confirming there was no significant difference among the groups in terms of the initial values of BHQ, demographic variables, and psychological scales.

**Table 1:** Initial values by groups.

	N	Control		N	Training A		N	Training B		N	Training C		ANOVA/chi-square test
		Mean	SD		Mean	SD		Mean	SD		Mean	SD	
GM-BHQ	39	101.667	7.339	16	101.602	9.409	15	100.754	6.949	12	102.877	4.588	F (3, 78) = 0.183, p = 0.908
FA-BHQ	39	100.976	3.198	16	99.484	3.782	15	100.895	3.233	12	100.242	3.495	F (3, 78) = 0.829, p = 0.482
Age	39	40.564	8.626	16	39.188	12.571	15	40.133	6.664	12	42.583	9.219	F (3, 78) = 0.315, p = 0.815
Sex	39	Male: 35; Female: 4		16	Male: 15; Female: 1		15	Male: 13; Female: 2		12	Male: 9; Female: 3		χ^2^ (3) = 2.521, p = 0.472
BMI	39	23.131	3.761	16	24.361	4.677	15	24.014	3.892	12	23.342	2.516	F (3, 78) = 0.478, p = 0.699
Interaction Anxiety	39	34.359	16.017	16	34.688	16.495	15	29.933	16.555				F (2, 67) = 0.459, p = 0.634
Exhaustion	39	15.564	5.911	16	19.875	6.602	15	16.267	5.457				F (2, 67) = 2.995, p = 0.057
Cynicism	39	11.538	4.978	16	12.500	5.203	15	13.467	7.405				F (2, 67) = 0.673, p = 0.513
Professional Efficacy	39	21.103	5.286	16	16.563	6.377	15	20.067	7.759				F (2, 67) = 3.128, p = 0.050
Work Engagement	39	54.923	16.865	16	44.563	16.415	15	55.000	18.944				F (2, 67) = 2.243, p = 0.114

Descriptive statistics of the participants and the correlation coefficients between the scales are shown in Table 2. Here, we excluded one of the data for ΔGM-BHQ (increase in GM-BHQ) because it showed a uniquely large figure dominating more than 10 percent of the pre-experiment GM-BHQ value. ΔGM-BHQ scores correlated with sex (r = −0.261, p < 0.05). On the other hand, ΔFA-BHQ (increase of FA-BHQ) scores correlated with Training A (r = 0.302, p < 0.05), ΔCynicism (increase of Cynicism. r = −0.244, p < 0.05), and ΔWork Engagement (increase of Work Engagement. r = 0.245, p < 0.05). Additionally, we wanted to include sex as another control variable in the models where the scores of the ΔGM-BHQ was used as the dependent variable.

**Table 2:** Descriptive statistics of the participants and correlation coefficients between the scales.

	Variable	Mean^a^	SD^a^	Mean^b^	SD^b^	1	2	3	4	5	6	7	8	9	10	11	12	13
1	Δinteraction Anxiety	1.243	8.381															
2	ΔExhaustion	1.171	4.791			0.045												
3	ΔCynicism	1.343	5.600			0.221	0.555^***^											
4	ΔProfessional Efficacy	1.143	4.378			−0.207	0.156	0.066										
5	ΔWork Engagement	0.300	13.261			−0.216	−0.216	−0.351^**^	0.228									
6	ΔGM-BHQ	−0.409	2.099	−0.664	2.313	0.057	−0.057	−0.035	0.035	0.066		−0.027	−0.152	−0.261^*^	0.059	0.118	0.181	−0.266^*^
7	ΔFA-BHQ	−0.465	1.700	−0.345	1.650	0.084	−0.195	−0.244^*^	0.055	0.245^*^	0.086		−0.055	0.085	0.086	0.302^**^	0.096	0.176
8	Age	40.157	9.201	40.512	9.187	0.092	0.039	0.074	0.061	−0.132	−0.077	−0.116		−0.009	0.153	−0.071	−0.020	0.094
9	Sex (male = 1, female = 2)	1.100	0.302	1.122	0.329	0.059	−0.032	−0.046	−0.055	0.021	−0.166	−0.011	−0.209		−0.307^**^	−0.089	0.016	0.162
10	BMI	23.601	3.989	23.563	3.798	0.122	−0.010	0.009	0.024	−0.065	0.004	0.106	0.213	−0.339^**^		0.104	0.056	−0.024
11	Training A	0.229	0.423	0.195	0.399	−0.073	−0.191	0.009	0.201	0.104	0.077	0.363^**^	−0.058	−0.068	0.104		−0.233^*^	−0.204
12	Training B	0.214	0.413	0.183	0.389	−0.141	−0.063	−0.164	0.047	−0.075	0.158	0.140	−0.001	0.058	0.054	−0.284^*^		−0.196
13	Training C			0.146	0.356													

Correlations for Control, Training A, and Training B (n = 70) appear below diagonal and correlations for Control, Training A, Training B, and Training C (n = 82) above diagonal.

*p < 0.05; **p < 0.01; ***p < 0.001. ^a^Mean/SD of Control, Training A, and Training B (n = 70); ^b^Mean/SD of Control, Training A, Training B, and Training C (n = 82).

The relations between initial values and its increments are shown in Table 3. ΔFA-BHQ (an increase of FA-BHQ) scores correlated with initial FA-BHQ scores (r = −0.245, p < 0.05), Likewise, all the increases in the psychological scale scores correlated with their initial scores (e.g., the correlation of Interaction Anxiety and ΔInteraction Anxiety (r = −0.381, p < 0.01)). Therefore, we decided to include all the initial scores of BHQs and psychological scales in the model individually as control variables in the following analyses.

**Table 3:** Initial value means and correlation coefficients with changes.

	Initial value mean	SD	Cronbach’sα	Correlation coefficient with its change
**Control**, **Training A**, **and Training B (n** = **70)**				
interaction Anxiety	33.486	16.111	0.937	−0.381^**^
Exhaustion	16.700	6.153	0.891	−0.478^***^
Cynicism	12.171	5.592	0.823	−0.455^***^
Professional Efficacy	19.843	6.312	0.877	−0.534^***^
Work Engagement	52.571	17.531	0.947	−0.248^*^
GM-BHQ	101.457	7.671		−0.117
FA-BHQ	100.617	3.354		−0.244^*^
**Control**, **Training A**, **Training B**, **and Training C (n** = **82)**				
GM-BHQ	101.665	7.297		−0.185
FA-BHQ	100.562	3.356		−0.245^*^

Table 4 (for Control, Training A, Training B, and Training C. n = 82) and Tables 5 (for Control, Training A, and Training B. n = 70) show the results of the regression analyses. In the model with ΔFA-BHQ (or ΔGM-BHQ) scores as the dependent variables, we entered the scores of the FA-BHQ (or GM-BHQ) at the time before the experiment (T1) as the control variable. Likewise, in the model with the increase in the psychological scales as the dependent variable, we entered the scores of the psychological scales at the time before the experiment (T1) as the control variables. As the main variables, we entered the dichotomous variables of Training A, Training B, and Training C in Step 1, and these training variables with ΔGM-BHQ in Step 2, and these training variables with ΔFA-BHQ in Step 3. As for the model using ΔGM-BHQ as the dependent variable, none of the variables significantly correlated with ΔGM-BHQ. On the other hand, in the model with ΔFA-BHQ as the dependent variable, Training A (R = 0.490, b = 0.393, p < 0.001), Training B (R = 0.490, b = 0.254, p = 0.019), and Training C (R = 0.490, b = 0.298, p = 0.006) significantly correlated with ΔFA-BHQ, indicating that the increase in FA-BHQ in those who participated a training irrespective of its kind was larger than in those who did not participate. In the model with ΔInteraction Anxiety as the dependent variable, only Training B (R = 0.455, b = −0.320, p < 0.006) significantly correlated with ΔFA-BHQ while Training A (R = 0.455, b = −0.058, p = 0.606) did not, indicating that interaction anxiety decreased only in those who participated a training with coaching.

**Table 4:** Multiple regression analysis of training, BHQ, and the psychological scales (for Control, Training A, Training B, and Training C).

	ΔGM-BHQ^a^				ΔFA-BHQ^a^	
	Step 1				Step 1	
	β^b^	p-value	β^b^	p-value	β^b^	p-value
**Control variables**						
Initial value	−0.167	0.124	−0.116	0.295	−0.183	0.074
Sex (male = 1, female = 2)			−0.197	0.081		
**Training variables**						
Training A	0.112	0.331	0.102	0.367	0.393	<0.001***
Training B	0.164	0.154	0.173	0.126	0.254	0.019*
Training C	−0.201	0.080	−0.173	0.131	0.298	0.006**
R	0.356	0.033*	0.403	0.019*	0.490	<0.001***
R^2^	0.127		0.162		0.240	

n = 82; *p < 0.05; **p < 0.01; ***p < 0.001.

^a^Changes between before and after the intervention.

^b^Standardized regression coefficient. Independent variables were selected using the forced entry method.

**Table 5:** Multiple regression analysis of training, BHQ, and the psychological scales (for Control, Training A, and Training B).

	ΔGM-BHQ^a^				ΔFA-BHQ^a^		ΔInteraction Anxiety^a^					
	Step 1				Step 1		Step 1		Step 2		Step 3	
	β^b^	p-value	β^b^	p-value	β^b^	p-value	β^b^	p-value	β^b^	p-value	β^b^	p-value
**Control variables**												
Initial value	−0.178	0.106	−0.119	0.288	−0.180	0.106	−0.355	0.002**	−0.399	<0.001***	−0.392	<0.001***
Sex (male = 1, female = 2)			−0.221	0.050								
**Training variables**												
Training A	0.164	0.145	0.145	0.190	0.404	<0.001***	−0.058	0.606	−0.135	0.253	−0.191	0.137
Training B	0.214	0.059	0.217	0.051	0.262	0.023*	−0.320	0.006**	−0.260	0.032*	−0.263	0.031*
**BHQ variables**												
ΔGM-BHQ									0.114	0.321		
ΔFA-BHQ											0.162	0.191
R	0.302	0.060	0.369	0.024*	0.477	<0.001***	0.455	0.001**	0.457	0.004**	0.463	0.003**
R^2^	0.091		0.136		0.227		0.207		0.209		0.214	
	Δ**exhaustion**^**a**^						Δ**Cynicism**^**a**^					
	**Step 1**		**Step 2**		**Step 3**		**Step 1**		**Step 2**		**Step 3**	
	**β**^**b**^	**p-value**	**β**^**b**^	**p-value**	**β**^**b**^	**p-value**	**β**^**b**^	**p-value**	**β**^**b**^	**p-value**	**β**^**b**^	**p-value**
**Control variables**												
Initial value	−0.456	<0.001***	−0.440	<0.001***	−0.470	<0.001***	−0.441	<0.001***	−0.432	<0.001***	−0.459	<0.001***
**Training variables**												
Training A	−0.092	0.431	−0.112	0.347	−0.018	0.891	−0.008	0.941	−0.019	0.870	0.120	0.327
Training B	−0.106	0.347	−0.176	0.132	−0.063	0.590	−0.112	0.330	−0.151	0.207	−0.033	0.773
**BHQ variables**												
ΔGM-BHQ			0.060	0.597					0.076	0.509		
ΔFA-BHQ					−0.161	0.180					−0.290	0.016*
R	0.493	<0.001***	0.493	0.001**	0.513	<0.001***	0.468	<0.001***	0.468	0.003**	0.535	<0.001***
R^2^	0.243		0.243		0.263		0.219		0.219		0.286	
	Δ**Professional Efficacy**^**a**^						Δ**Work Engagement**^**a**^					
	**Step 1**		**Step 2**		**Step 3**		**Step 1**		**Step 2**		**Step 3**	
	**β**^**b**^	**p-value**	**β**^**b**^	**p-value**	**β**^**b**^	**p-value**	**β**^**b**^	**p-value**	**β**^**b**^	**p-value**	**β**^**b**^	**p-value**
**Control variables**												
Initial value	−0.514	<0.001***	−0.529	<0.001***	−0.512	<0.001***	−0.281	0.019*	−0.232	0.071	−0.264	0.031*
**Training variables**												
Training A	0.077	0.496	0.084	0.465	0.094	0.454	0.035	0.775	0.027	0.833	−0.108	0.436
Training B	0.078	0.471	0.094	0.399	0.088	0.437	−0.136	0.255	−0.051	0.694	−0.129	0.307
**BHQ variables**												
ΔGM-BHQ			−0.087	0.426					0.031	0.804		
ΔFA-BHQ					−0.038	0.748					0.300	0.023*
R	0.542	<0.001***	0.548	<0.001***	0.543	<0.001***	0.333	0.043*	0.258	0.347	0.371	0.045*
R^2^	0.294		0.300		0.295		0.111		0.066		0.137	

n = 70; *p < 0.05; **p < 0.01; ***p < 0.001.

^a^Changes between before and after the intervention.

^b^Standardized regression coefficient. Independent variables were selected using the forced entry method.

Step 1 includes control and training variables only. Step 2 includes control, training, and GM-BHQ variables. Step 3 includes control, training, and FA-BHQ variables.

**Table 6:** Multiple comparison of the adjusted-mean ΔFA-BHQ scores and ΔInteraction Anxiety.

	Control	Training A	Training B	Training C	ANCOVA
ΔFA-BHQ					
Non-adjusted mean	−1.099	0.660	−0.014	0.351	
SD	1.515	1.720	1.434	1.148	
Adjusted mean^d^	−1.062	0.563	0.016	0.322	F (3, 77) = 6.075, p < 0.001***
p-value of multiple comparisons		<0.001^0-1^***	0.019^0-2^*	0.006^0-3^**	
			0.310^1-2^	0.671^1–3^	
				0.595^2‐3^	
ΔInteraction Anxiety					
Non-adjusted mean	1.900	0.125	−6.235		
SD	8.708	8.213	17.644		
Adjusted mean^e^	2.007	0.384	−6.732		F (2, 69) = 4.106, p = 0.021*
p-value of multiple comparisons		0.606^0-1^	0.006^0-2^**		
			0.058^1-2^		

n = 82 for ΔFA-BHQ.

n = 70 for ΔInteraction Anxiety.

*p < 0.05; **p < 0.01; ***p < 0.001.

^d^adjusted for scores of FA-BHQ at the time before the experiment (T1) by ANCOVA method.

^e^adjusted for scores of Interaction Anxiety at the time before the experiment (T1) by ANCOVA method.

^0–1^p-value derived from the comparison between “Control” and “Training A” by ANCOVA method.

^0–2^p-value derived from the comparison between “Control” and “Training B” by ANCOVA method.

^0–3^p-value derived from the comparison between “Control” and “Training C” by ANCOVA method.

^1–2^p-value derived from the comparison between “Training A” and “Training B” by ANCOVA method.

^1–3^p-value derived from the comparison between “Training A” and “Training C” by ANCOVA method.

^2–3^p-value derived from the comparison between “Training B” and “Training C” by ANCOVA method.

ANCOVA, analysis of covariance.

On the other hand, in the model with ΔCynicism or ΔWork Engagement as the dependent variable, only ΔFA-BHQ (R = 0.535, b = −0.290, p = 0.016; R = 0.371, b = 0.300, p = 0.023) significantly correlated with these variables but Training A (R = 0.535, b = 0.120, p = 0.327; R = 0.371, b = −0.108, p = 0.436) or Training B (R = 0.535, b = −0.033, p = 0.773; R = 0.371, b = −0.129, p = 0.307) did not, indicating that a decrease in cynicism or increase in work engagement may be observed only in those who showed an increased FA-BHQ. However, in the model with ΔExhaustion or ΔProfessional Efficacy as the dependent variables, none of the independent variables significantly correlated with these variables.

**Figure 2:**
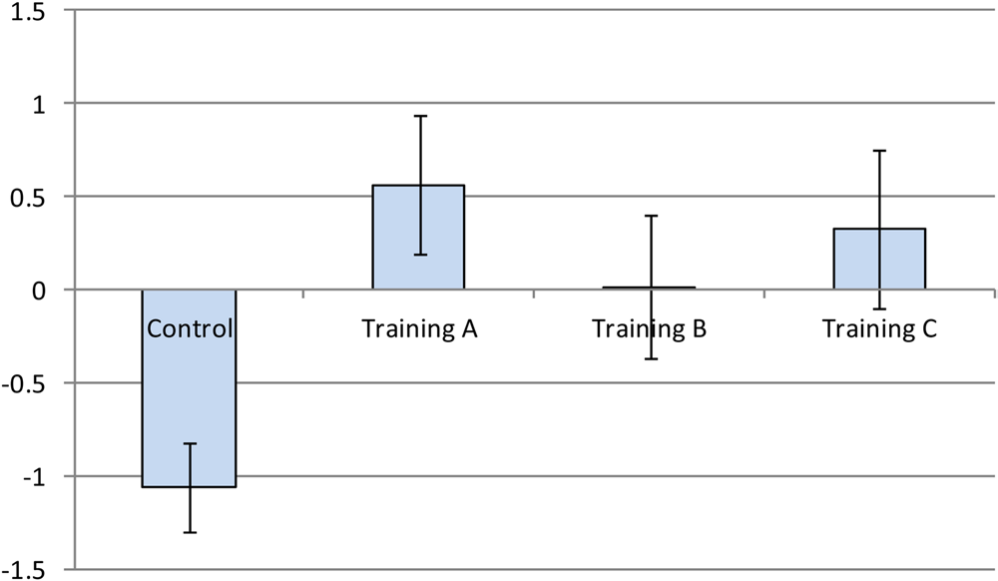
Multiple comparison of the adjusted-mean ΔFA-BHQ scores. Error bars indicate the standard error of the mean (SEM).

**Figure 3:**
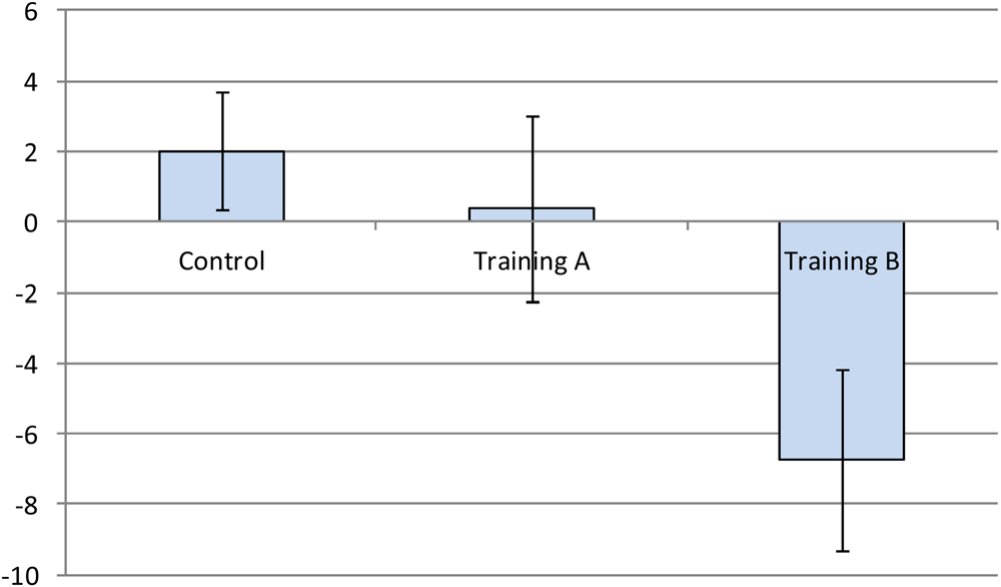
Multiple comparison of the adjusted-mean ΔInteraction Anxiety. Error bars indicate the standard error of the mean (SEM).

Additionally, in Table 6, multiple comparisons of the adjusted-mean of the ΔFA-BHQ scores with analysis of covariance (ANCOVA) revealed significant differences in the Control with Training A (p < 0.001), Training B (p = 0.019), and Training C (p = 0.006), with a lower score of the Control. Besides, multiple comparisons using adjusted-mean ΔInteraction Anxiety scores with ANCOVA found significant differences between Control and Training B (p = 0.006), with lower scores of Training B. Therefore, the findings are consistent with those of the regression analysis in Table 5. The results are also shown in Figs. 2 and 3.

In summary, training had a positive correlation with an increase in FA-BHQ scores after adjusting for the scores of the FA-BHQ at T1. In turn, an increase in FA-BHQ scores had a positive correlation with increases in Work Engagement and a negative correlation with increases in Cynicism after adjusting for the scores of Work Engagement and Cynicism at T1, respectively. Besides, training accompanied by coaching had a positive correlation with decreases in Interaction Anxiety after adjusting for the scores of Interaction Anxiety at T1. However, training/coaching had no significant correlation with increases/decreases in GM-BHQ, Work Engagement, Cynicism, Exhaustion, and Professional Efficacy. Likewise, an increase in FA-BHQ (or GM-BHQ) scores had no significant correlation with increases/decreases in Interaction Anxiety, Exhaustion, and Professional Efficacy.

## Discussion

**Figure 4:**
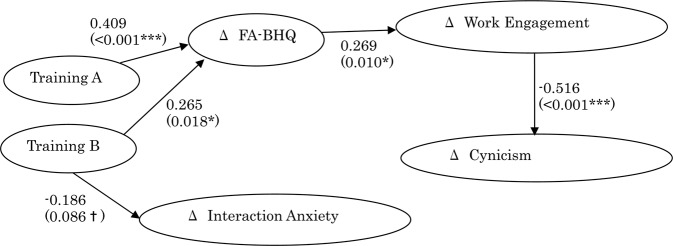
Elucidated association between human resource development trainings, FA-BHQ, and Work Engagement (and Interaction Anxiety and Cynicism). Goodness-of-fit indices: χ^2^ = 20.063; *df* = 23; RMSEA = 0.000; PCLOSE = 0.807; GFI = 0.942; AGFI = 0.887; NFI = 0.848; CFI = 1.000. Covariances between variables and initial values of each variable are omitted from the figure above (available upon request).

Over the past two decades, the number of studies on work engagement has increased rapidly. Work engagement refers to a positive, affective-motivational state of high energy combined with high levels of dedication and a strong focus on work^1^ leading to various work-related outcomes including higher work performance^2–6^. Several studies have indicated that training or coaching could increase work engagement^2,19,20,22^, but other studies have shown contradicting results^21,26^. These inconsistencies may partly be due to indirectness between training/coaching and the work engagement. Another concern is that work engagement has been inconsistently understood by different researchers; i.e., although it is currently most representatively defined to be composed of vigor, dedication and absorption^56^, in the past it was simply assessed as the opposite of burnout^69^. Moreover, work engagement is known to be strongly associated with internal communication^66,67^. Therefore, we investigated the relationship between training and brain structure as well as between the brain structure and work engagement (and related but different psychological scales) in cognitively normal participants. Brain structure was assessed using neuroimaging-derived measures including GM-BHQ and FA-BHQ, which are approved as the international standard (H.861.1) by ITU-T. Work Engagement was assessed using the Utrecht Work Engagement Scale developed by Schaufeli *et al*.^56^. To validate and enrich the analysis, we employed another two representative questionnaires, which are known to be close to but different from work engagement: The Social interaction Anxiety Scale^57^ and the Maslach Burnout Inventory-General Survey^58^ to gauge the levels of human relation ineffectiveness and burnout, respectively. The latter scale is subdivided into three variables including “Exhaustion,” “Cynicism,” and “Professional Efficacy.” The results of the present study indicate that training is associated with an increase in FA-BHQ scores, and an increase in FA-BHQ scores is associated with an increase in Work Engagement and a decrease in Cynicism. On the other hand, training with coaching was associated with a decrease in Interaction Anxiety. However, no correlation was observed for training with Work Engagement or the subscales of Burnout. Likewise, no correlation was observed for FA-BHQ with Exhaustion, Professional Efficacy, and Interaction Anxiety. The elucidated associations are depicted in Fig. 4. For reference, the figures of the standardized path coefficient controlled by the scores at the time before the experiment (T1), calculated using AMOS Version 26 (IBM Corp., Armonk, NY, USA), are also shown.

The figures are the standardized path coefficient controlled by the scores at the time before the experiment (T1). Only statistically significant (*p < 0.05; **p < 0.01; ***p < 0.001) and marginally significant (^†^p < 0.10) paths are shown and p-values are displayed in parentheses. Correlations between variables are not shown but are available upon request.

As for Work Engagement, the result is supportive of our hypothesis. Training may increase brain health measured by FA-BHQ, and indirectly increase Work Engagement if it is mediated by an increase in FA-BHQ. However, training did not have a direct effect on Work Engagement due to the indirectness between them. Although the correlation between some kind of training and FA has been elucidated in previous research^34–38^, the novelty of our study lies in the significant association noted between HRDT and brain health. We may attribute the current result to emotional and cognitive mechanisms within the brain. The former is, as previous research regarding integrative body-mind mediation training suggests, the one responsible for the enhanced white-matter integrity in the anterior cingulate cortex (ACC)^36^, which is part of a network implicated in the development of self-regulation and indicated as one of the regions associated with strong stress^73^. It is reasonable to assume that HRDT in this experiment also contributed to an increase in self-regulation and a decrease in stress, and in turn an increased FA-BHQ within the ACC and related regions. The latter is because any complex task requires transmission of information through a series of distant cortical regions with distinct task-relevant functions^74^ and therefore changes in white matter, including axon diameter, the number of myelinated axons in a tract, the thickness of myelin, or other morphological features such as internodal distance, determine the speed of impulse propagation and thus could contribute to increased functional performance with learning^74,75^. It is possible to assume that HRDT in the current research was perceived as a sufficiently complex task by the participants to alter task-related regions influencing the scores of FA-BHQ.

No changes in GM-BHQ after HRDT in the current research were not expected but consistent with the results of previous research by Tang and colleagues^36^. One possible explanation for this result is that the methods used to detect alterations in FA and gray matter may have had different sensitivities. Or it is also possible that the training may have resulted in changes in both FA and gray matter, but with different time courses. We plan to study this possibility in future experiments.

The association between brain health and work engagement has also, to our knowledge, never been elucidated except for some related studies which indicated that cognitive ability is among the strongest predictors of training effectiveness^28^ because it helps to prioritize organizational goals, multitasking^29^, engaging in training, and performing the skills taught in the course^30–33^. Therefore, our results are in line with those of previous research, and at the same time add new information about the mediating role of FA-BHQ between HRDT and work engagement.

Employee cynicism has several negative consequences, including reduced levels of performance, job satisfaction, and organizational commitment, and increased levels of intention to quit^76,77^. Previous research indicates that training performed as a socialization process provided participants with adequate information about the system change and therefore reduced uncertainty, fear, and cynicism^78,79^. However, contradictory to these results, another study by Collier and colleagues^80^ looking at cynicism and humanism in internal medicine residents across the United States showed that sixty-one percent of the 4128 residents become more cynical and 23% become less humanistic during medical training due to uncontrollable intrinsic mechanisms including how trainees perceived trainers and trainings. These inconsistencies may also be attributed to the distance between training and cynicism. Our result indicates that cynicism may be lowered only when brain health becomes better and work engagement becomes higher as a result of training. For reference, it is indicated that the relation between FA-BHQ and Cynicism is perfectly mediated by Work Engagement as is shown in Fig. 4. This may be because burnout is a chronic state that results from accumulated experiences of overload^81^. Specifically, cynicism, a distant attitude toward the job^81^, may not only appear during the work but also when telling others about one’s job^82^. Work engagement, however, can be rather short-lived and emerges in situations where sufficient resources, including good supports from peers, are present^82^. This result is consistent with the work by Taris and his colleagues, which found that work engagement could be a leading factor in burnout^50^.

Unexpectedly, our results showed no significant association between increases in FA-BHQ and Interaction Anxiety, a finding which contradicts the results of previous research. For instance, recent DTI studies have shown that reduced FA within the uncinate fasciculus (UF), the most prominent fiber tract linking the amygdala and orbitofrontal cortex, is associated with lower levels of extroversion and fewer friends^83^ and higher levels of suspiciousness and interpersonal difficulties^84^ in schizotypal personality disorder. Moreover, an association between UF FA and impulsivity and aggression in schizophrenia has previously been observed^85^. In line with this, a previous study found WM abnormality within UF in individuals with generalized social anxiety disorder, reflecting the aberrant patterns of amygdala, frontal, or their interactions in response to social threat in this disorder^86^. However, the common feature impossible to overlook of these studies is that the participants were people with brain-related diseases and different from those in the current research which used cognitively normal adults as the participants. Therefore, the current results might be altered if we used subjects with any disorder.

Another unexpected but reasonable result was the lack of an association between increases in FA-BHQ and Professional Efficacy because the results of previous studies have shown that self-efficacy can have either a positive^87,88^ or a negative^89–92^ effect on performance. Since such an inconsistency has not been observed in studies involving Work Engagement, it may be better to deal with Professional Efficacy and Work Engagement as different scales in contrast with the discussion that the engagement could be regarded as the opposite of MBI^70,71^. In support of this hypothesis, a recent meta-analysis of the within-person self-efficacy literature concluded that self-efficacy is not the driving force compelling higher performance; rather, it is an indicator of whether people have succeeded in the past^93^. Therefore, our result is consistent with these studies and indicates the sequential possibility that professional efficacy may be heightened after executing higher performances, which are demonstrated by increased work engagement.

As is shown in Fig. 2, the significant difference in increases of FA-BHQ between the Control group and the Training group was not only because the scores of the latter increased but also because the scores of the former decreased. Therefore, we cannot say that “training may increase FA-BHQ” but can say “training may prevent a decrease in FA-BHQ.” Then, what is the reason for the decrease in the Control group? One possible reason may be the seasonal changes in brain volume. Indeed, previous studies have shown that brain mass in the common shrew decreases 10–26% from summer to winter and regrows 9–16% in spring, with a decrease in the hypothalamus, thalamus, and hippocampal volume and a later regrowth in the spring, whereas the neocortex and striatum volumes decrease in winter and do not recover in size^94^. The period during which the current research was done was from September to December, which represent the seasons during which brain volume decreases.

Lastly, we would like to discuss the reasons for the contradicting results regarding Training B, i.e., the training accompanied by coaching. The result that Training B was only associated with a decrease in interaction anxiety indicates that coaching is most effective in alleviating anxiety about human relations. However, we have to note that Training B, at the same time, had a marginally weaker association with an increase in FA-BHQ than other kinds of training without coaching, contradicting the results of previous research which found a positive effect of coaching^2^. In a business setting, coaching is generally seen as a means of developing people to enable more effective performance and fulfillment of potential^95^ because coaching is one of the most powerful methods of developing soft skills^96^. However, as with any development intervention, it does not necessarily work successfully for all individuals and in all situations^97^ because there is an inconsistent relationship between coaching and coaching outcomes due to several factors against successful coaching attributable to both coachees and coaches^25,26,79^. For instance, Kilburg^98^ identified several factors in coachees (e.g., lack of motivation, unrealistic expectations, lack of follow-up) and coaches (e.g., insufficient empathy, lack of expertise in the area of concern, poor techniques) that play against a successful coaching outcome. In line with this, Crabb^27^ insisted that the coach may need to manage their own emotions during conversations by remaining objective and free from bias, which could prove to be difficult, because highly motivated employees engage in more self-regulated learning than less motivated employees, ultimately enhancing training/coaching effectiveness^33^. Thus, any mismatch between participants and coaches may have lowered the autonomous motivation of participants and in turn moderated the increase in FA-BHQ. Therefore, the result of the current research provides the possibility to use brain information to evaluate training/coaching effectiveness from the viewpoint of neuroscience as there is no objective measure to evaluate the effect of training/coaching except ROI. ROI is calculated by subtracting the costs of training/coaching from the estimated value of the outcomes of the training/coaching^99^ but there are many different applications of this formula reported in the literature (e.g., deliberately underestimating the financial return figure and producing a conservative estimate) making the metric unreliable^26,100,101^.

This study has five limitations. First, the association between brain health and actual work-related behaviors was not examined in this study, and an illustration of the association of brain health with actual behaviors may have improved the validity of the results. Second, the sample size was small in this study, and studies with a larger sample size may have an increased generalizability of the results. Third, it is not clear if the level of brain condition and work engagement raised by the intervention can be maintained on a long-term basis. A chronological survey that will show what kind of persons can maintain the standard will make a significant contribution to academia. Fourth, a variety of factors, including coach and coachee compatibility, can have an impact on the consequences of coaching reducing anxiety but less improving brain or work engagement in the current study. An analysis that puts the personalities of coaches and coachees as mediation parameters can further evolve the study. Fifth, this study is for Japanese people, and it is necessary to verify whether it applies  to other countries. For example, previous studies have shown that organizational commitment, a psychological measure close to work engagement, has a significant correlation with fatigue, a psychological measure close to burnout, in China, but not in the Philippines^102,103^. Therefore, future studies are needed to explore the relationship between FA-BHQ and actual behaviors using larger sample sizes by chronological ways using more diverse questionnaires for more various nationalities to discern the mechanisms.

### Ethics Statement

The studies involving human participants were reviewed and approved by the Ethics Committees of Kyoto University (approval number 27-P-13) and Tokyo Institute of Technology (approval number A16038). The patients/participants provided their written informed consent to participate in this study.

## Data Availability

The raw data supporting the conclusions of this manuscript will be made available by the authors, without undue reservation, to any qualified researcher.
